# Retrograde Open Mesenteric Stenting Failure Requiring Aorto-Mesenteric Bypass in Acute-on-Chronic Mesenteric Ischemia: A Case Report

**DOI:** 10.70352/scrj.cr.26-0479

**Published:** 2026-11-10

**Authors:** Lahcen Kaddouri, Amine Bouallala, Emna Benabdelaziz, Nazih Chaouch

**Affiliations:** Department of Vascular Surgery, Cannes Hospital - Simone Veil, Cannes, France

**Keywords:** mesenteric ischemia, stenting of the superior mesenteric artery, stent thrombosis, aorto-mesenteric bypass

## Abstract

**INTRODUCTION:**

Chronic mesenteric ischemia (CMI) is a rare pathology, most commonly associated with multivessel atherosclerosis, whose diagnosis is often delayed due to nonspecific symptoms. Endovascular treatment is the first-line treatment option, although its results may be limited by restenosis or intrastent thrombosis. Open surgery remains an important option in cases where endoluminal treatment has failed. The aim of this paper is to illustrate, through a clinical case, the diagnostic and therapeutic challenges associated with severe CMI.

**CASE PRESENTATION:**

A 69-year-old female patient, a long-term smoker being treated for peripheral arterial disease, presented with postprandial abdominal pain that had been present for 6 months, accompanied by severe malnutrition. The CT angiogram revealed occlusion of the celiac trunk and the superior mesenteric artery, with the development of extensive collateral circulation. Initial endovascular revascularization via stenting was performed in a patient presenting with gastrointestinal distress that had required segmental resection of necrotic ileojejunal loops. The course of the disease was characterized by a recurrence of symptoms and stent thrombosis, leading to surgical revascularization via an aorto-mesenteric bypass. The postoperative course was favorable, with the mesenteric angina gradually subsiding, significant weight gain, and lasting functional improvement.

**CONCLUSIONS:**

This case highlights the limitations of endovascular treatment alone in severe multivessel disease and confirms the value of a personalized treatment strategy incorporating open surgery to optimize long-term outcomes.

## INTRODUCTION

Chronic mesenteric ischemia (CMI) is a pathological process that develops over a prolonged period, most commonly as a secondary consequence of atherosclerosis. It is difficult to obtain accurate epidemiological estimates, as not all patients are correctly diagnosed during their lifetime. It is estimated that fewer than 1 in 100000 hospital admissions are related to CMI. Due to the presence of collateral networks, the clinical manifestations of CMI generally appear when at least 2 of the 3 mesenteric vessels are either occluded or have severe stenosis.^1)^

Under normal physiological conditions, a hyperemic response occurs immediately after a meal: the resulting vasodilation enables the intestinal organs to meet the increased oxygen demand associated with digestion. In patients with superior mesenteric ischemia, this hyperemic response is insufficient to meet these requirements due to the presence of a stenosis or atherosclerotic occlusion^2)^; this is known as mesenteric angina.

This leads to the development of abdominal symptoms, as well as an increased risk of progression to gangrene or intestinal necrosis in the event of further acute deterioration.

Over the past few decades, endovascular treatment, particularly stenting, has become the treatment of choice for CMI. However, in some cases, endovascular stenting may fail, whether due to restenosis, stent migration, or persistent occlusion, leading to clinical deterioration that then requires open surgery.

We report the case of a 69-year-old woman who presented with acute-on-CMI. She had a 6-month history of postprandial abdominal pain suggestive of CMI and was admitted with acute intestinal ischemia complicated by bowel necrosis requiring resection. She was initially treated with hybrid endovascular revascularization (retrograde open mesenteric stenting; ROMS), followed by an aorto-mesenteric bypass after failure of the endovascular treatment.

## CASE PRESENTATION

We report the case of a 69-year-old woman with a history of stenting of the right renal artery, peripheral arterial disease, and a history of 50 pack-years of active smoking, with no history of smoking cessation. She was admitted for an acute-on-CMI, presenting as postprandial abdominal pain that had been progressing for 6 months. This cramp-like pain following meals, combined with early satiety, had led to a gradual reduction in food intake, resulting in marked weight loss and a current weight of 32 kg.

On admission, the patient was emaciated, with normal blood pressure, a soft abdomen, mild abdominal tenderness, and no rigidity. Laboratory tests revealed mild normochromic, normocytic anemia, with a major inflammatory syndrome (C-reactive protein [CRP] of 291 mg/L), elevated lactate levels, hypoalbuminemia, hypophosphatemia, and hypocalcemia, as well as normal renal function.

Abdominal computed tomography angiography (CTA) demonstrated severe multivessel mesenteric occlusive disease involving all 3 mesenteric arteries. The celiac trunk and the superior mesenteric artery (SMA) were occluded at their origins. Moderate atherosclerotic calcification was noted at the origins of the celiac trunk and SMA. The SMA occlusion extended for approximately 40 mm, with distal reconstitution and preserved distal opacification through a well-developed collateral network, indicating the presence of a suitable distal target for revascularization. CTA also demonstrated bowel wall thickening involving the ascending colon and distal ileal loops, associated with minimal free fluid in the right paracolic gutter; findings consistent with acute bowel ischemia superimposed on chronic mesenteric arterial occlusive disease. No preoperative conventional angiography was performed, as CTA provided sufficient anatomical information to assess the feasibility of mesenteric revascularization and to plan the surgical strategy (**Fig. 1**).

**Fig. 1:**
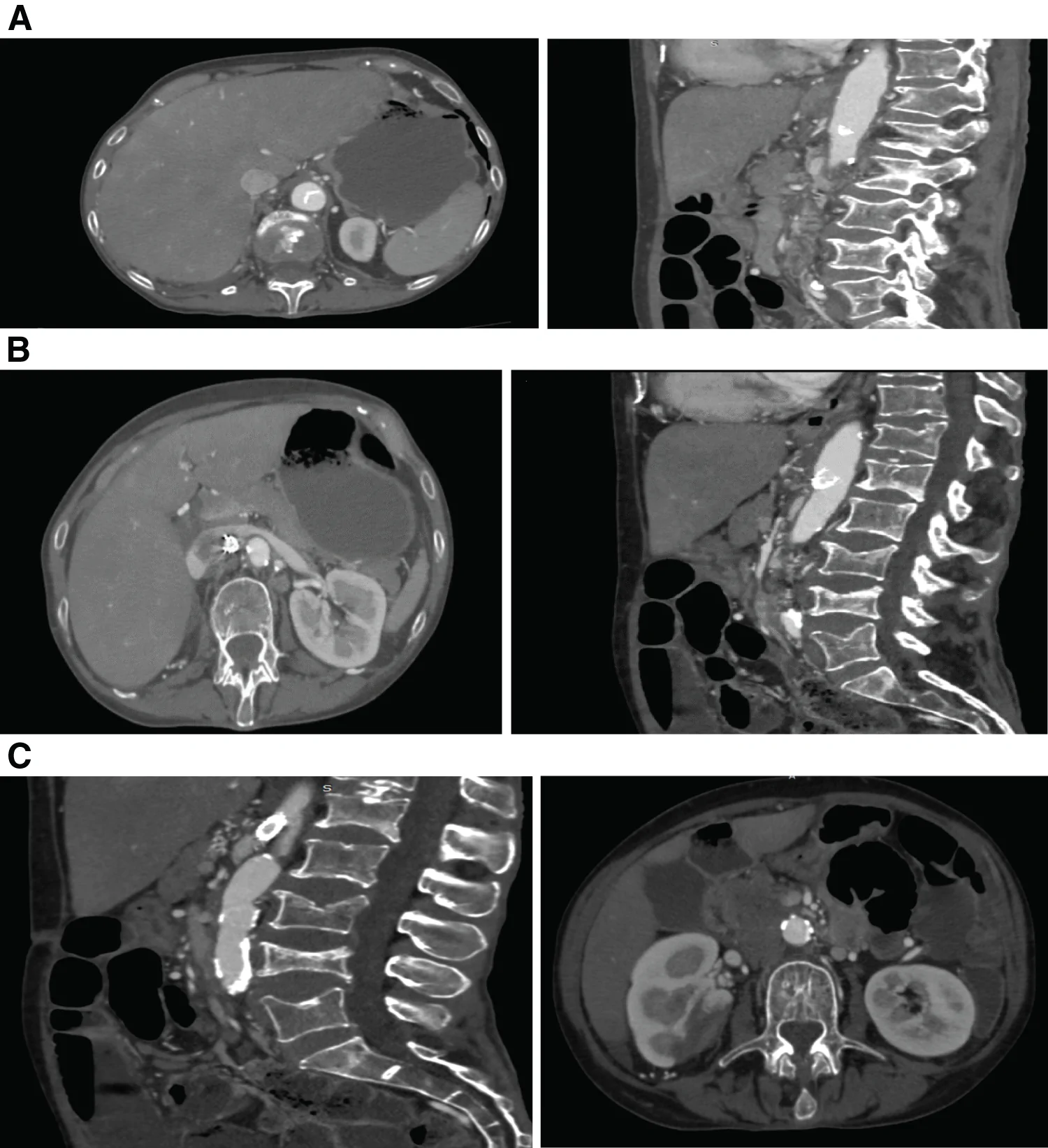
Preoperative CTA demonstrating multivessel mesenteric arterial occlusive disease. (**A**) Sagittal and axial CTA images demonstrating flush occlusion of the celiac trunk at its origin, associated with marked ostial atherosclerotic calcification. (**B**) Sagittal and axial CTA images demonstrating flush occlusion of the SMA at its origin, with distal reconstitution through a well-developed collateral network, indicating a suitable distal target for revascularization. (**C**) Sagittal and axial CTA images demonstrating occlusion of the IMA at its origin, with distal reconstitution through collateral circulation. A previously implanted right renal artery stent remains patent. CTA, computed tomography angiography; IMA, inferior mesenteric artery; SMA, superior mesenteric artery

The multidisciplinary consultation recommended exploratory abdominal surgery, which revealed segmental necrosis of the terminal ileal loops and necrotic changes in the jejunum, justifying the performance of a revascularization procedure as the first-line treatment.

Conventional antegrade endovascular recanalization was considered technically unfeasible because of the flush ostial occlusion of the SMA, which precluded antegrade catheterization. Although a primary mesenteric bypass was considered, bowel necrosis requiring intestinal resection and the contaminated intra-abdominal operative field were deemed to increase the risk of prosthetic graft infection. Consequently, hybrid revascularization by ROMS was selected as the initial treatment strategy. After exposing the artery during a laparotomy, an arteriotomy was performed, allowing for retrograde catheterization. Angioplasty was then carried out using a 6-mm covered stent. Intraoperative assessment revealed good patency of the device from the ostium. The procedure was completed by closing the arteriotomy using an arterial patch (**Fig. 2**). Subsequently, a resection of the terminal ileum and ascending cecum was performed due to ischemic lesions of the intestinal walls of mesenteric origin. The remaining length of the small intestine was estimated at 180 cm. A terminating ileostomy was created in the right iliac fossa, in conjunction with a terminating colostomy in the right subcostal region (**Fig. 3**).

**Fig. 2:**
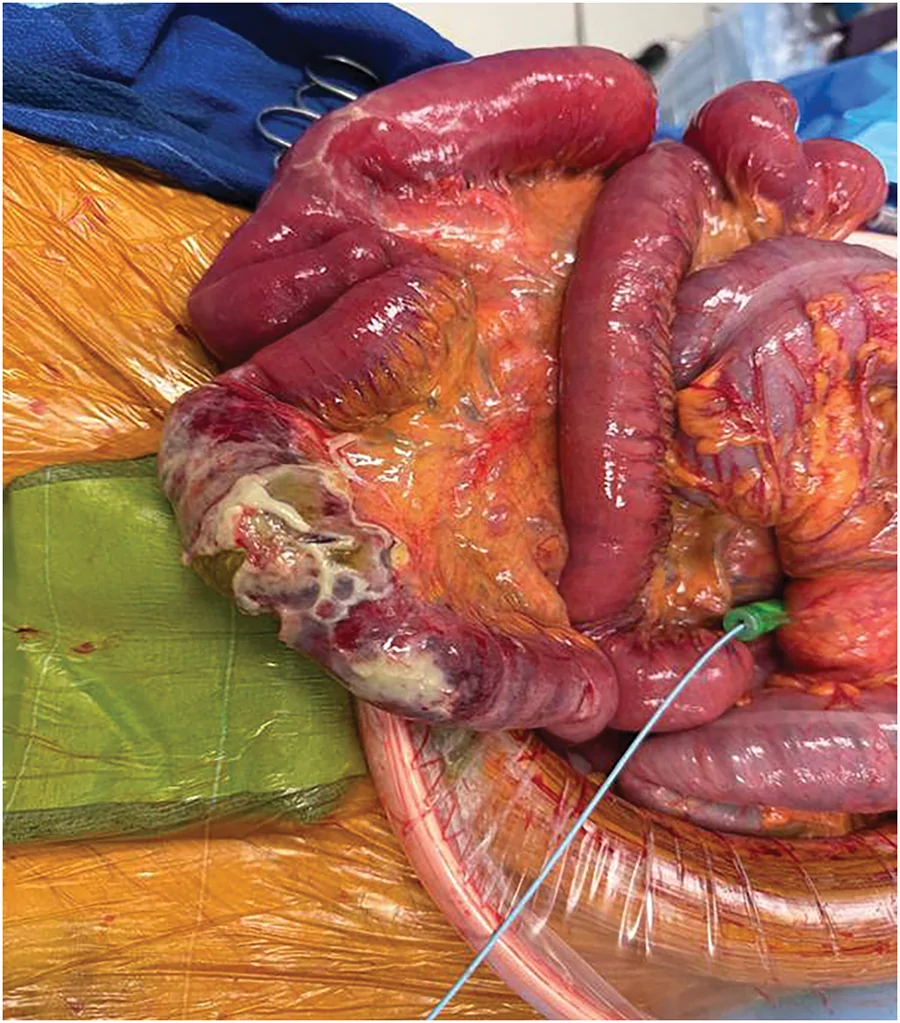
Intraoperative view showing the placement of an introducer into the SMA using the open retrograde mesenteric revascularization (ROMS), in conjunction with segmental necrosis of the terminal ileal loops and a necrotic appearance of the jejunum. ROMS, retrograde open mesenteric stenting; SMA, superior mesenteric artery

**Fig. 3:**
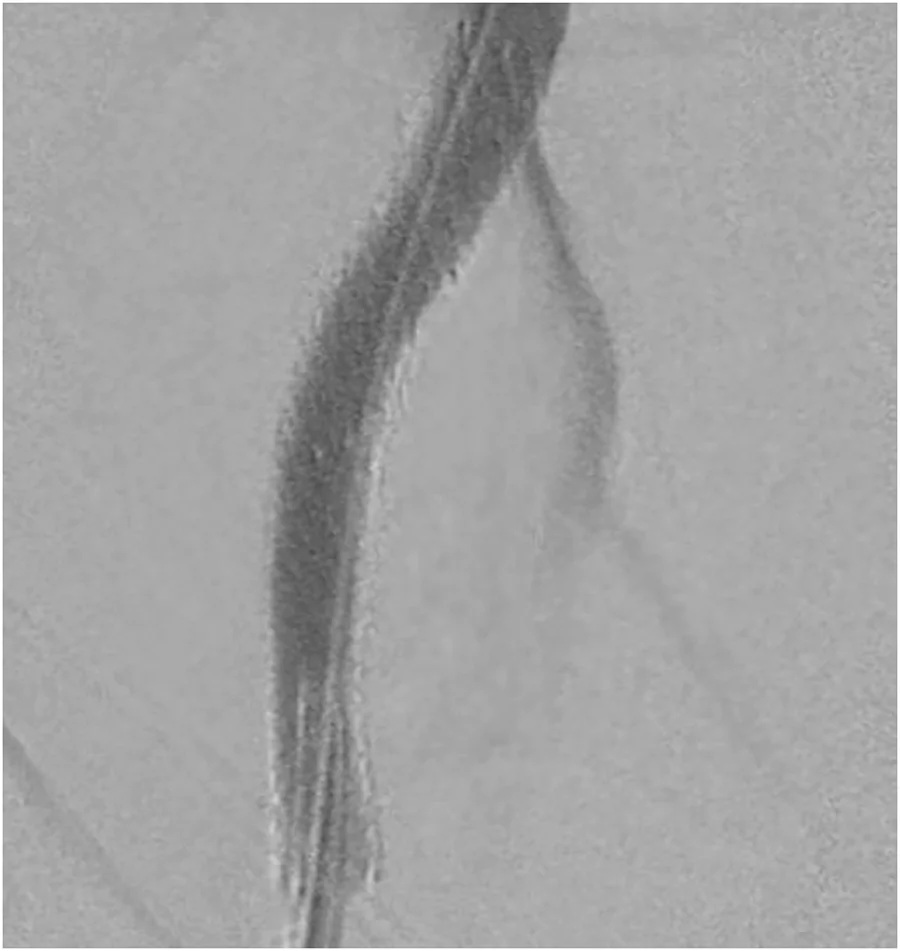
Arteriographic assessment using ROMS. ROMS, retrograde open mesenteric stenting

Six months after ROMS, the patient presented with recurrent postprandial abdominal pain, which brought her weight to 29 kg. CTA demonstrated thrombosis of the SMA stent (**Fig. 4**). The patient had poor adherence to the prescribed medical therapy and continued heavy smoking despite repeated smoking cessation advice. Although the exact mechanism of stent thrombosis could not be determined, these factors may have contributed to stent failure, together with the severity of the underlying diffuse atherosclerotic disease. In view of the stent occlusion and recurrent mesenteric ischemia, the multidisciplinary team decided to perform definitive open revascularization with an aorto-mesenteric bypass. This involved retracting the small intestine to the right, and the left lobe of the liver after its release, followed by dissection of the supra-celiac aorta with clamping for 45 minutes. After assessment and clamping of both renal arteries, the mesenteric artery, and the primary iliac arteries, a 2-cm longitudinal arteriotomy was made at the level of the celiac aorta, followed by the placement of an 8-mm polytetrafluoroethylene (PTFE) graft and anastomosis at the upper end via an end-to-side suture using 2 hemistitches. The graft was then tunnelled pre-pancreatically and trans-mesocolically, followed by an lower end-to-side anastomosis onto the SMA trunk (**Figs. 5** and **6**).

**Fig. 4:**
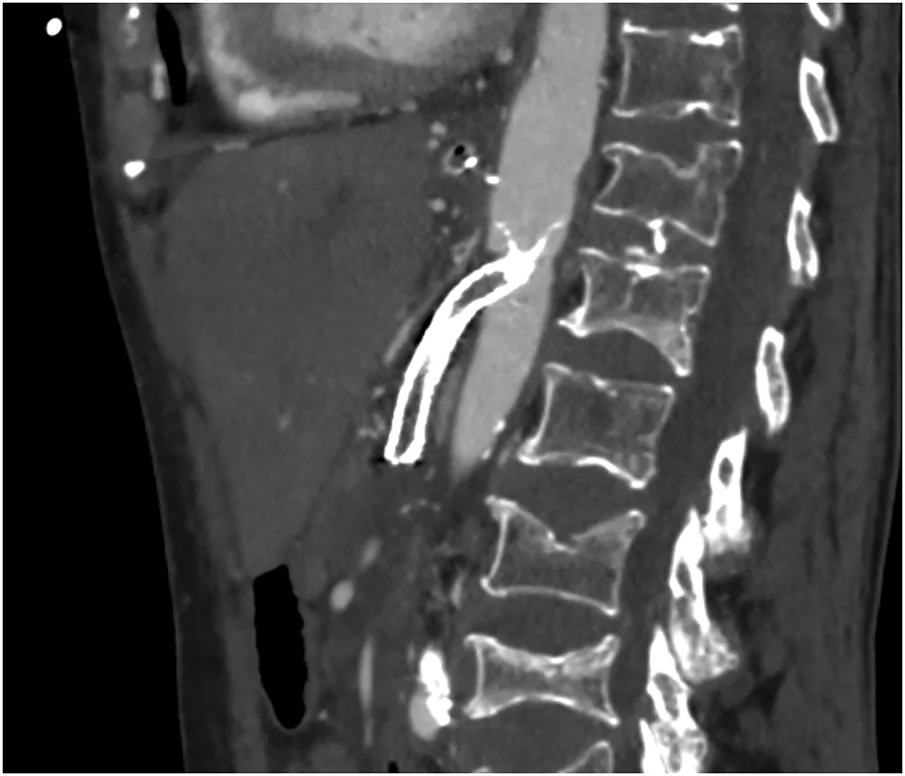
Sagittal section of a CT angiogram of the abdominal aorta showing thrombosis of the SMA stent. SMA, superior mesenteric artery

**Fig. 5:**
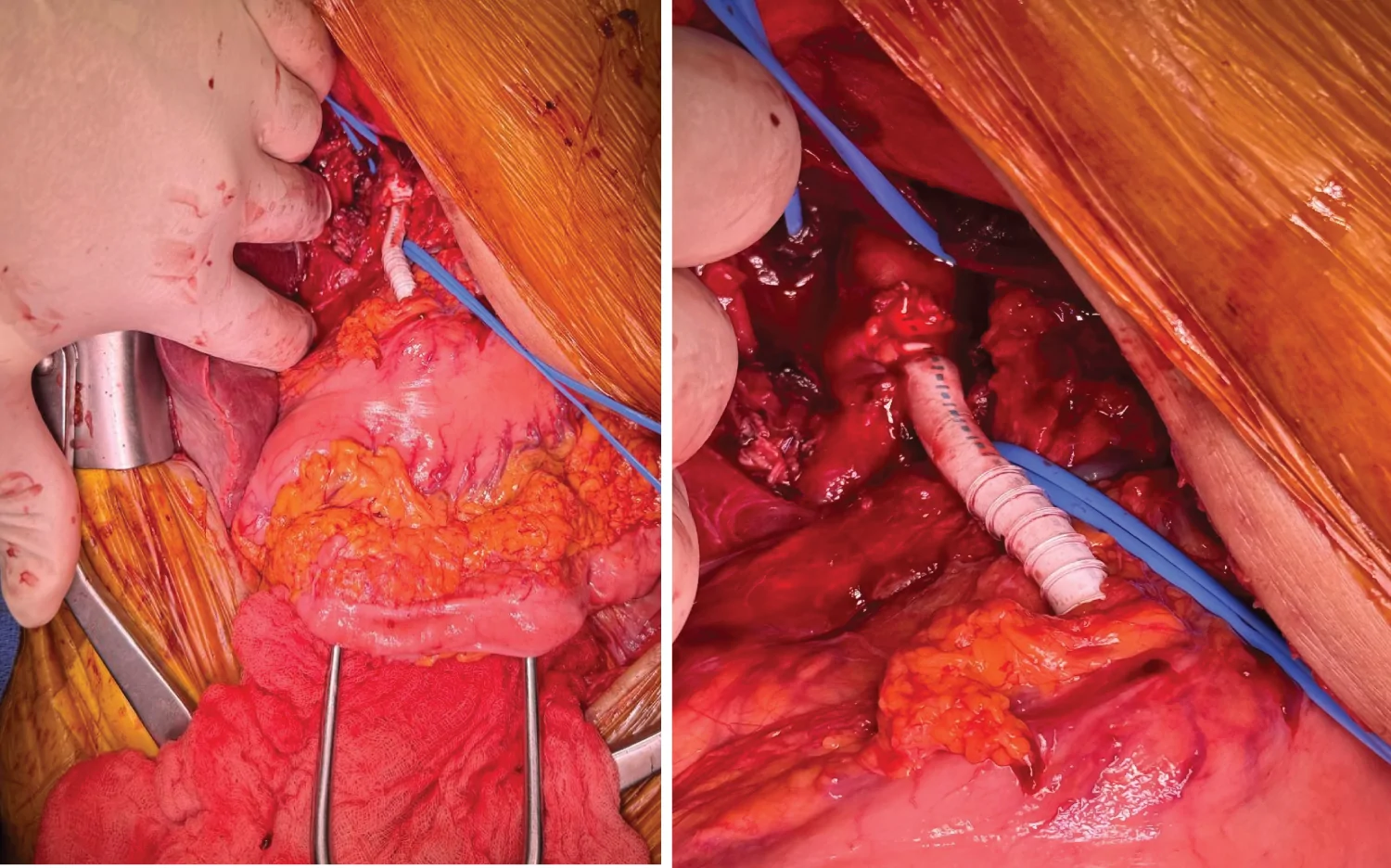
Intraoperative view showing the creation of an aorto-mesenteric bypass via a subduodenal approach in a case of complicated CMI. CMI, chronic mesenteric ischemia

**Fig. 6:**
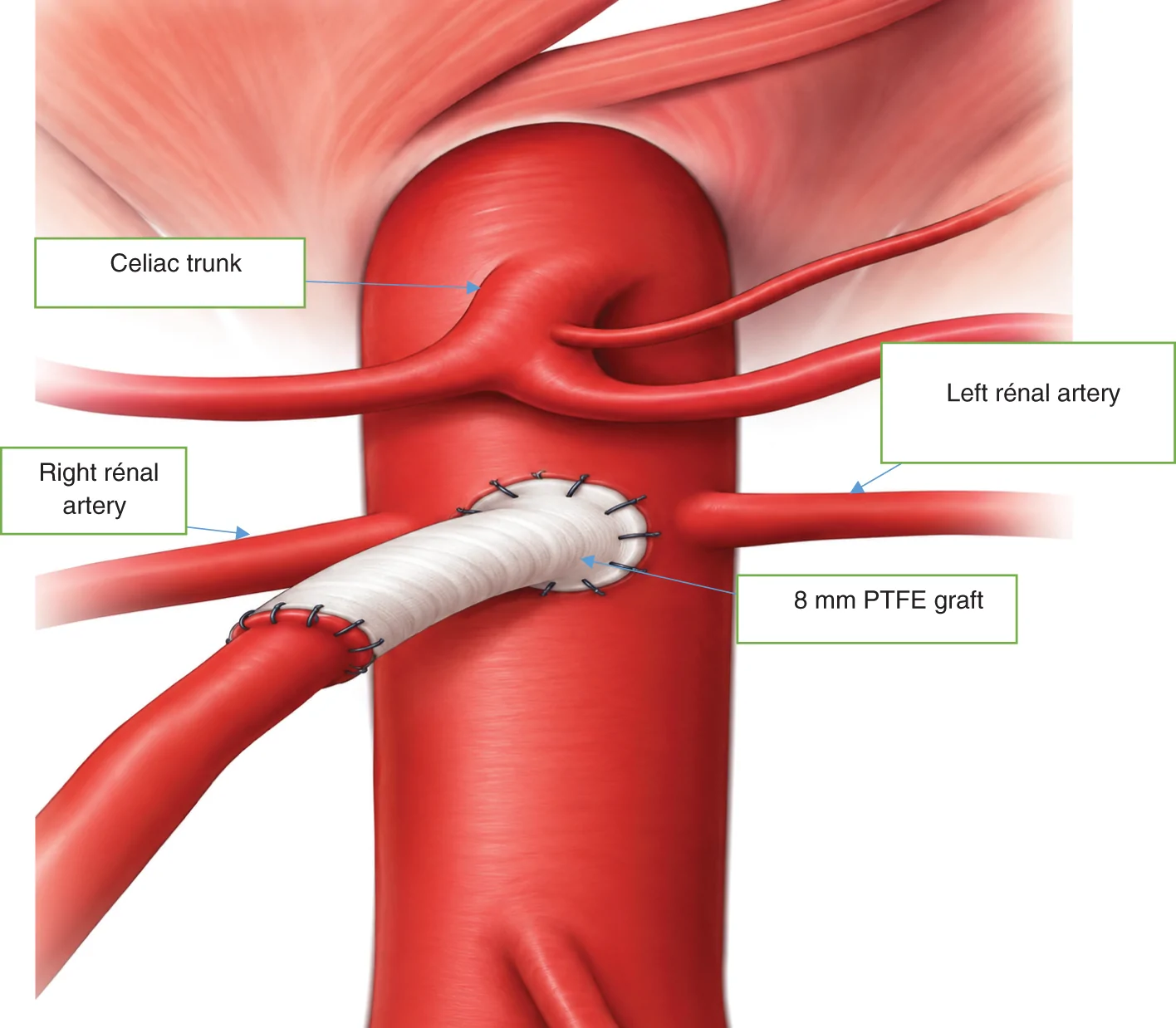
Aorto-mesenteric bypass using an 8-mm PTFE graft: a surgical technique. PTFE, polytetrafluoroethylene

The postoperative course was uneventful from both a hemodynamic and gastrointestinal perspective. The patient experienced gradual clinical improvement, with complete resolution of postprandial abdominal pain within the first few weeks after surgery.

Oral feeding was progressively reintroduced and was well tolerated, resulting in an improvement in nutritional status. Ileostomy output was satisfactory, with no evidence of severe short-bowel syndrome. Restoration of intestinal continuity was planned following nutritional optimization. However, the patient was subsequently lost to follow-up; therefore, no long-term clinical or imaging assessment of graft patency, nutritional status, or stoma outcome was available (**Table 1**).

**Table 1:** Timeline of the patient’s clinical course according to the CARE guidelines.

Time	Clinical event
6 months before admission	Postprandial abdominal pain, progressive weight loss
Admission (Day 0)	Acute-on-CMI diagnosed by CTA
Day 0	Exploratory laparotomy, bowel resection, ROMS with covered SMA stent
Postoperative period	Clinical improvement and hospital discharge
6 months	Recurrent postprandial pain; CTA showed SMA stent thrombosis
6 months	Aorto-mesenteric bypass with 8-mm PTFE graft
Early postoperative follow-up	Resolution of symptoms, nutritional improvement
Later follow-up	Lost to follow-up

CMI, chronic mesenteric ischemia; CTA, computed tomography angiography; ROMS, retrograde open mesenteric stenting; SMA, superior mesenteric artery

## DISCUSSIONS

CMI was first described in 1918 by Goodman under the name “abdominal angina.”^3)^ Its onset is gradual, and diagnosis is often delayed. Treatment of the underlying lesion(s) is essential to prevent the onset of acute mesenteric ischemia, which can lead to intestinal infarction and death

It states that a necessary condition for the manifestation of mesenteric angina is significant involvement affecting at least 2 of the 3 main feeding arteries. The remaining one-third demonstrates that this condition is not always sufficient, as evidenced by cases of asymptomatic significant 3-trunk lesions. Celiac trunk dysgenesis, which is very rare, is an exception to this figure. Angina attacks only occur in cases of advanced global lesions that significantly reduce the overall blood supply from the 3 arterial trunks. The minimum pathological threshold for vascular compromise, based on arteriographic data, is established at approximately 70%.^4)^

The current study sheds new light on this common misconception.^5)^ Risk factors that predispose a patient to atherosclerosis include smoking, high blood pressure, diabetes mellitus, and high cholesterol.^6)^

This study reports a higher incidence of CMI of atherosclerotic origin and median arcuate ligament syndrome in female patients and confirms the female predisposition reported by other studies.^7,8)^

In symptomatic patients with a single-vessel atherosclerotic stenosis of the mesenteric artery, the location of the stenosis appeared to be significant, as CMI was present in 40.6% of patients with severe stenosis of the SMA, but in only 5.6% of patients with severe stenosis of the celiac trunk.^9)^

Asymptomatic patients with CMI are managed conservatively with antiplatelet therapy and smoking cessation. Symptomatic CMI warrants revascularization. Revascularization can be performed surgically or via minimally invasive endovascular procedures. Endovascular options such as percutaneous transluminal angioplasty with or without stenting have more favorable outcomes and are now used as first-line treatment in specialist centers.^10)^ Stenting is generally performed if residual stenosis persists after angioplasty. Although percutaneous transluminal angioplasty with stenting is limited by an increased risk of restenosis, the use of covered stents offers exceptional patency rates.^11)^ According to the European Society for Vascular Surgery, routine stenting of the mesenteric artery, as opposed to simple balloon angioplasty, is recommended in patients with CMI requiring endovascular treatment. The advantage of using drug-eluting stents over bare-metal stents for the treatment of atherosclerotic CMI has been a subject of debate, with conflicting results in 2 retrospective studies. In a study involving 225 patients, drug-eluting stents were associated with a lower restenosis rate, a lower rate of recurrence of clinical symptoms, and fewer reinterventions compared with bare-metal stents (for all endpoints, approximately 8% versus 50%).^11)^

A less invasive approach to the ROMS technique could improve morbidity and mortality. This hybrid technique uses a midline laparotomy to directly expose the mesenteric artery and allow retrograde endovascular access. Milner et al.^12)^ performed the first technically successful ROMS, exposing the SMA at the root of the mesentery and performing a direct puncture. As in our case, they observed no procedure-related complications, and the patient initially recovered well (before dying of multiple organ failure despite a patent SMA stent). Wyers et al. exposed the SMA and used a patch angioplasty technique to treat 6 patients via ROMS. They achieved a technical success rate of 100% and a periprocedural mortality rate of 17%. The mortality rate for ROMS was lower than that for traditional surgical revascularization (80%).^13)^

Although ROMS has demonstrated excellent technical success and acceptable short- and mid-term outcomes, late stent failure remains an important limitation. As summarized in **Table 2**, reports describing ROMS failure requiring secondary intervention remain scarce. In the series by Wyers et al.^13)^, no patient required open bypass, although 1 underwent repeat endovascular stenting for restenosis. Oderich et al.^14)^ reported excellent secondary patency after reintervention, with only 1 patient requiring open aorto-SMA bypass. More recently, Cirillo-Penn et al.^15)^ described 1 patient who underwent retrograde mesenteric bypass following covered stent failure. In comparison, our patient developed complete covered stent thrombosis 6 months after ROMS and required definitive aorto-mesenteric bypass, highlighting that open reconstruction remains an essential salvage option after endovascular failure (**Table 2**).

**Table 2:** Published reports of ROMS failure and subsequent secondary intervention.

Study	Patients (ROMS)	Stent type	Antithrombotic therapy	Time to stent failure	Mechanism of failure	Secondary treatment	Patency/outcome
Cirillo-Penn et al., 2023^15)^	18	Balloon-expandable covered stent	Aspirin + clopidogrel	7 months	Stent failure requiring surgical revision	Retrograde mesenteric bypass	Primary patency 87.5% at 2 years
Present case	1	Balloon-expandable covered stent	Aspirin + clopidogrel for 8 weeks	6 months	Stent occlusion/failure	Retrograde mesenteric bypass	Early clinical improvement; long-term follow-up unavailable
Oderich et al., 2018 ^14)^	54	Balloon-expandable stents (36 bare-metal, 18 covered)	Lifelong aspirin + clopidogrel for 4–8 weeks	Mean 8 months	Restenosis	Aorto-SMA bypass (1 patient)	Secondary patency 90% at 2 years
Wyers et al., 2007^13)^	6	Balloon-expandable bare-metal stents	Aspirin indefinitely + clopidogrel for 30 days	Not reported (during mean follow-up 13 ± 7 months)	Asymptomatic recurrent SMA restenosis	Repeat endovascular stenting (1 patient); no open bypass	Primary patency 50%

ROMS, retrograde open mesenteric stenting; SMA, superior mesenteric artery

In cases of atherothrombotic acute mesenteric ischemia (AMI), when open surgical revascularization is chosen, the 2 techniques available are ROMS and bypass surgery. In recent years, ROMS has become the hybrid surgical technique of choice for atherothrombotic AMI requiring exploratory laparotomy, combining open surgical techniques with endovascular techniques. ROMS is performed via laparotomy, with an approach to the mesenteric artery similar to that used in embolectomy. Direct retrograde puncture of the mesenteric artery allows retrograde catheterization of the mesenteric artery toward the aorta, as close as possible to the lesion. This technique has several advantages as it allows exploration of the small intestine via laparotomy and, compared to a bypass, it enables faster vascular repair with comparable patency rates, whilst avoiding aortic clamping as well as graft coverage and infection issues. The most commonly used stents are covered steel stents deployed over a balloon.^16)^ If catheterization fails, a bypass is always an option. Mesenteric bypasses are the last-resort revascularization technique for the SMA.

Surgical intervention is generally indicated in cases of intestinal ischemia. It can be performed using either an antegrade (from the supra-celiac aorta) or retrograde (from the iliac artery) approach, using a vein or a prosthetic graft to bypass one or more vessels.^17)^

## CONCLUSIONS

This case highlights the limitations of endovascular treatment alone in severe, multivessel atherosclerotic mesenteric occlusive disease presenting with acute-on-CMI and confirms the need for an individualized treatment strategy, incorporating hybrid techniques and open surgery, to optimize mesenteric revascularization and improve functional outcomes.

## DECLARATIONS

### Availability of data and materials

The data supporting the findings of this case report are available from the corresponding author upon reasonable request.
